# Epidemiological profile trends and cost of sickle cell disease in Brazil from 2008 to 2022

**DOI:** 10.1590/acb403025

**Published:** 2025-03-31

**Authors:** Luiza Telles, Paulo Henrique Moreira Melo, Gabriele Eckerdt Lech, Luana Baptistele Dornelas, Natália Zaneti Sampaio, Ayla Gerk, Madeleine Carroll, Cristina Camargo

**Affiliations:** 1 Instituto de Educação Médica – Rio de Janeiro (RJ) – Brazil.; 2 Universidade Federal de Minas Gerais – Faculdade de Medicina – Belo Horizonte (MG) – Brazil.; 3 Pontifícia Universidade Católica do Rio Grande do Sul – Porto Alegre (RS) – Brazil.; 4 Santa Casa de São Paulo – Faculdade de Ciências Médicas – São Paulo (SP) – Brazil.; 5 Universidade de Araraquara – Araraquara (SP) – Brazil.; 6 Harvard University – Harvard Medical School – Program in Global Surgery and Social Change – Boston (MA) – United States.; 7 McGill University – Department of Surgical and Interventional Sciences – Montreal (QC) – Canada.; 8 Montreal Children’s Hospital – Harvey E. Beardmore Division of Pediatric Surgery – Montreal (QC) – Canada.; 9 Universidade de São Paulo – Faculdade de Medicina – Laboratory of Microsurgery and Plastic Surgery – São Paulo (SP) – Brazil.

**Keywords:** Public Health, Anemia, Sickle Cell, Global Health, Cost of Illness

## Abstract

**Purpose:**

This study aimed to evaluate the epidemiological profile trends and economic impact of sickle cell disease (SCD) in Brazil from 2008 to 2022, focusing on incidence, mortality, and healthcare costs.

**Methods:**

A cross-sectional analysis was conducted using data from the Fundação Oswaldo Cruz’s platform, Plataforma de Ciência de Dados Aplicada à Saúde, encompassing hospitalizations related to SCD from January 2008 to December 2022. The International Classification of Diseases codes for SCD were used to retrieve data on incidence, mortality, procedures performed, and healthcare costs.

**Results:**

The study included 151,535 hospitalizations for SCD, with 69.92% associated with SCD crises and 22.48% without crises. The mean annual hospitalizations were higher for crises (6,883.06) compared to those without crises (2,221.12). Mortality rates were significantly higher for patients hospitalized with crises compared to those without crises (*p* < 0.001). The economic impact of SCD was substantial, with annual costs exceeding 413 million USD.

**Conclusion:**

This study revealed a significant burden of SCD in Brazil, characterized by high hospitalization rates, particularly among younger patients, and elevated mortality rates associated with crises. Prospective studies and public health interventions are warranted to address SCD and mitigate its impact on public health.

## Introduction

There are approximately two million carriers of sickle cell disease (SCD) in Brazil. Approximately 25,000 to 50,000 individuals carry the homozygotic form (HbSS) of the disease, which represents 3,500 live births annually^1^^–^ 4. SCD impacts a range of 60,000 to 100,000 Brazilians, exhibiting prevalence of 45.92 cases per 100,000 live births documented between 2015 and 2019^5^. Earlier investigations have approximated that SCD correlates with a mortality rate spanning from 0.12 to 0.54 per 100,000 individuals^5^^–^ 8.

The economic impact of SCD is high in Brazil (413,639,180 USD yearly)^5^. The vaso-occlusive crisis emerged as the predominant complication, incurring the highest annual cost, amounting to an estimate of 11,400,410 USD for the adult population annually^5^. It is important to note that the standard of care and chronic wound care complications are managed and impact direct costs^5^.

However, a closer analysis of Brazilian population data regarding the prevalence by region, periods of crisis, economic impact of SCD, and mortality is needed. This study’s primary objective was to evaluate national trends in incidence, mortality, and public health system costs regarding SCD in Brazil from 2008 to 2022.

## Methods

This cross-sectional study evaluated the national annual rates of SCD in Brazil. Data was retrieved from January 2008 to December 2022, from the Fundação Oswaldo Cruz (Fiocruz)’s platform, Plataforma de Ciência de Dados Aplicada à Saúde (PCDaS)9. PCDaS is a national, open-access Brazilian discharge database that provides health-related information for patients admitted to the Universal Health System (SUS), which includes public and private health systems. We accessed the SUS Hospitalar Healthcare Information System, in chapter XIX of this platform. We collected data using the International Classification of Diseases (ICD-10), chapter III, codes for Sickle Cell Trait (D57), Sickle Cell Disease (D57.1), and Sickle Cell Crisis (D57.0). The annual estimates collected were incidence by sex, age, Brazilian regions, procedures performed in those patients, and cost related to SCD and intensive care unit (ICU) admissions. The mortality rate was also assessed.

### Statistical analysis

#### Data representation

This investigation presented data considering the nature and distribution of variables. Parametric data was summarized using mean and standard deviation, non-parametric data by median and interquartile range, and proportions by percentage.

#### Inferential analysis

For inferential analysis, the study employed a time-series analysis utilizing time-series graphics, and AutoRegressive Integrated Moving Average (ARIMA) was employed for regression analysis.

#### Cost analysis

To analyze the economic dimensions, hospital costs were categorized in US dollars as follows: 0–100, 100–500, 500–1,000, 1,000–5,000, and > 5,000.

### Statistical parameters

The study adhered to a significance level (alpha) of 5%, with a study power of 80%. Statistical analyses were performed using STATA v18 (StataCorp., Stata Statistical Software: Release 18, College Station, TX, United States of America).

### Ethical considerations

The study used publicly accessible secondary data online from the Fiocruz database, PcDaS, and adhered to the International Guidelines for the Development of Research Involving Human Subjects. Consequently, it is exempt from formal ethical procedures.

## Results

### Overall results

We collected data on 151,535 hospitalizations due to SCD from 2008 to 2022. Among these cases, 105,957 (69.92%) were associated with SCD with crises (ICD-10 D57.0), while 34,060 (22.48%) were associated with SCD without crises (ICD-10 D57.1). An additional 11,174 (7.75%) cases of other SCD were excluded from this study (Table 1).

**Table 1:** Sickle cell disease (SCD) and falcemic crisis admissions by age.

Age (years old)	SCD admissions	SCD crises admissions
20–25	2,909	16,318
25–30	2,501	14,732
30–35	2,255	13,897
35–40	2,100	12,765
40–45	1,945	11,983
45–50	1,800	11,200
50–55	1,700	10,570
55–60	1,550	9,860
60–65	1,250	8,987
65–70	1,100	7,930
70–75	135	489
75–80	143	445
80–85	115	360
85–90	63	226
+90	41	140

Source: Elaborated by the authors.

Both hospitalizations for sickle cell anemia with crisis and those for sickle cell anemia without crises exhibited a slight predominance of the male population: 50.18% were female patients *versus* 51.18% male patients.

**Figure 1:**
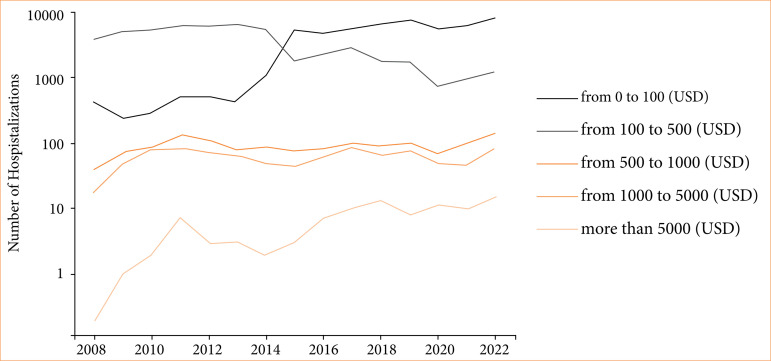
Annual trends in hospitalizations of SCD with crisis costs across different price categories.

SCD: sickle cell disease; USD: United States dollar. Source: Elaborated by the authors.

**Figure 2:**
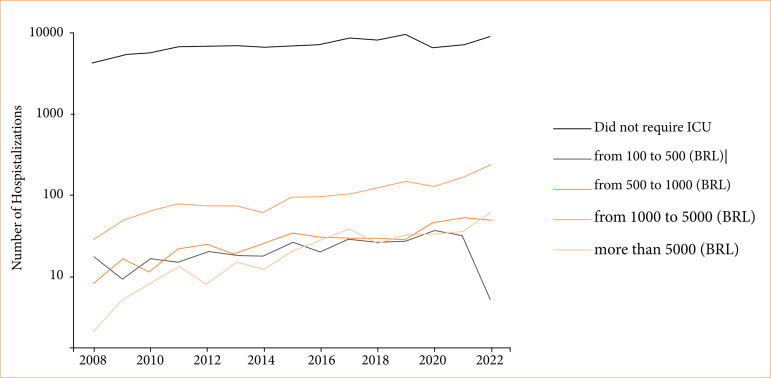
Annual trends in ICU bed costs of SCD with crisis across different price categories.

BRL: Brazilian real; ICU: intensive care unit; SCD: sickle cell disease. Source: Elaborated by the authors.

The statistical examination revealed that the mean annual hospitalizations for sickle cell anemia with crisis were 7,063.8, with a 95% confidence interval (95%CI) of 6,883.06 ± 374.6 (1,498.36–6,084.6) (Fig. 1). Similarly, for sickle cell anemia without crisis, the mean annual hospitalization count was 2,221.1 ± 115.5, with a 95%CI spanning from 462.0–1,974.9 (Fig. 2).

Concerning the types of procedures that were performed, both SCD with crises and without crises were the same. Hemolytic anemia treatment was the main procedure performed, respectively 97,306 and 30,402. It was followed by diagnosis or urgent care treatment in pediatrics, and urgent care treatment in general. When analyzing the surgical delivery, 115 evaluations occurred in urgent surgical care, 91 splenectomies were performed, and 29 multiple surgeries and 17 exploratory laparotomies performed.

### Sickle cell disease by age

When analyzing the data according to the age of the patients, SCD with and without crisis was most prevalent among 15 to 20 years old, presenting 17,051 and 3,612 cases respectively.

### Sickle cell disease by regions

Concerning the regions, the Southeast reached the biggest number of SCD for both with and without crisis, presenting 53,811 and 22,062 cases, respectively. The South achieved the lowest number in both cases, with 5,047 cases of SCD with crisis and 1,109 without crisis.

### Economic impact of sickle cell disease

**Figure 3:**
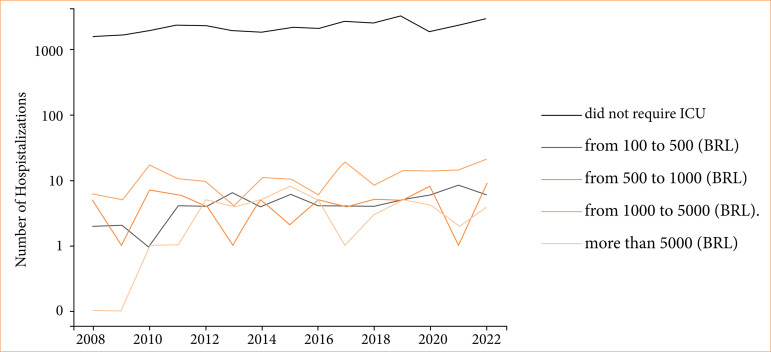
Annual trends in ICU bed costs of SCD without crisis across different price categories.

BRL: Brazilian real; ICD: intensive care unit; SCD: Sickle cell disease. Source: Elaborated by the authors.

The costs of ICU stays were analyzed. In terms of conversion, we considered 1 USD equivalent to R$ 5. Among hospitalizations that required intensive care treatment, the average ICU annual costs for each such hospitalization ranged from R$ 1,910.91 to R$ 2,977.21 (around $ 382.18 to $ 595.44) (Figs. 2 and 3). In a time analysis, these patients had higher costs in ICU in 2021 and 2022 (Fig. 1). Costs from SCD patients without a crisis in the ICU were lower–from around $ 343.85 to $ 563.85.

### Mortality of sickle cell disease

Concerning mortality, our study showed an average of 76 deaths per year among patients hospitalized with crisis, with a 95%CI between 74.5 ± 5.4 (21.6–62.9). For those without crisis, the mean annual death count was 21.2, with a 95%CI ranging from 20.87 ± 1.5 (6.04–17.65).

**Figure 4:**
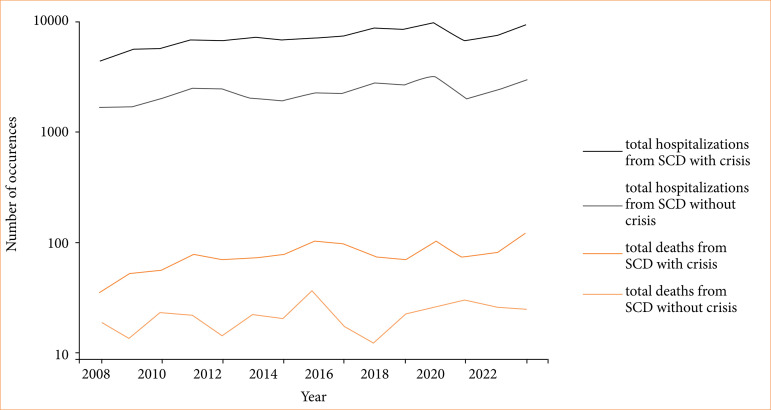
Hospitalizations and deaths from SCD.

The statistical significance of the differences in hospitalization and mortality rates for cases with crises compared to those without crisis was confirmed with a *p* < 0.001. This level of significance, well below the 0.01% threshold, robustly suggests that the observed differences are not due to random variation but represent a genuine disparity in the data. The results of hospitalizations and deaths from SCD are registered in Fig. 4.

SCD: sickle cell disorder. Source: Elaborated by the authors.

A time series regression method known as ARIMA revealed a significant relationship between patients hospitalized for sickle cell anemia with crises and mortality, with *p* = 0.004, 95%CI 11.6–59.36, and coefficient = 35.5. In contrast, no significant relationship was found between hospitalizations for sickle cell anemia without crisis and mortality. A *p* = 0.015, 95%CI = 6.5–60.4, and coefficient = 33.4 showed that there was a strong link between hospitalizations of patients with anemia and death.

## Discussion

In our analysis, out of 151,353 hospitalizations due to SCD, 69.92% were associated with sickle cell anemia with crisis, and 22.48% were associated with sickle cell anemia without crise. In both groups, most patients were male. It is also worth reinforcing that the mean annual hospitalization for the group with crisis was higher (6,883.06) than the group without crisis (2,221.12). As to mortality, the first group had an average of 76 deaths per year and the second group had an average of 21.2 deaths per year. We address higher mortality rates for a higher number of patients in SCD crisis.

Regarding the geographical disparities in hospitalizations due to SCD, the majority is located in the Brazilian Southeast and the minority in the South. Although we do not have enough data to understand this disparity, we hypothesized that these results reflect the higher number of centers in the Southeast, which promote migration from the other Brazilian’s region to Southeast region.

Concerning cost analysis, we found that ICU annual costs ranged from R$ 1,910.91 to R$ 2,977.21 in patients with crisis and without a crisis in the ICU diminished to the costs from $ 343.85 to $ 563.85. However, research conducted by Silva-Pinto et al.5 has found that, in Brazil, the annual SCD cost was more than 413 million USD. The same study found that, for acute complications, the standard care average costs were 1,835 USD and 987 USD for adults and children, respectively. As to chronic complications, the average costs were 769 USD and 116 USD^5^. These figures suggest a possible explanation for the findings of our study, which observed that the average costs of hospitalizations for anemia with crisis, i.e., anemia with acute complications, were higher than for anemia without crisis. The treatment for acute complications, such as blood transfusions and splenectomy, among others, is more costly than the management of chronic complications of the disease^5^^,^ 10 ^–^ 12.

In the last few years, only a very small number of studies about SCD in Brazil have been published. In this context, our study is fundamental to exposing data on this disease, which is relatively frequent in Brazil, and also its associated conditions. We looked over the last 15 years of data on hospitalizations in the whole country due to SCD and its consequences on anemia with or without crisis. However, this study has its limitations. First, since the data used for the analysis is from a public database and depends on the classification of diseases and procedures, the total number of patients of interest may be underestimated due to mistakes in classification by the hospitals, which can lead to errors in published data. Second, using only hospitalizations might not reflect the real incidence of the disease due to the possibility of ambulatorial treatment. Third, we need to consider the excluded data about other sickle cell disorders, which may influence the generalization of the results.

Our analysis serves as a foundation for prospective studies and the formulation of public health strategies. To prevent SCD crises and subsequently mitigate mortality rates and public health expenditures, enhancing patient care is imperative. To accomplish this objective, the management of SCD in Brazil needs a multidisciplinary approach, characterized by enhanced access to hydroxyurea therapy, the implementation of standardized and evidence-based pain management protocols, utilization of prophylactic antibiotics, execution of vaccination strategies, and the integration of genetic counseling and new therapies. Furthermore, key components of a more effective management strategy encompass patient education initiatives, as well as the incorporation of telemedicine and remote monitoring technologies. These measures collectively contribute to an improved standard of care and align with our overarching goal of minimizing the impact of the SCD crisis on public health.

## Conclusion

This study suggested that younger SCD patients are being hospitalized in Brazil and higher mortality rates are associated with SCD crisis. Beyond that, we concluded that SCD exerts a substantial economic impact, with an annual cost exceeding 413 million USD. To improve SCD patients’ outcomes, we encourage new studies on standardized and evidence-based pain management protocols.

## Data Availability

The data is public available in the DATASUS platform.
